# Investigating biological mechanisms of adverse birth outcomes and early child development in Amhara, Ethiopia: protocol of biospecimen collection and analysis of the Enhancing Nutrition and Antenatal Infection Treatment (ENAT) randomised effectiveness study

**DOI:** 10.1136/bmjopen-2024-098686

**Published:** 2025-04-28

**Authors:** Unmesha Roy Paladhi, Firehiwot Workneh, Estifanos Baye, Mulatu Melese Derebe, Kalkidan Yibeltal, Nebiyou Fasil, Sophie Driker, Fred Van Dyk, Theresa I Chin, Krysten North, Sarah K G Jensen, Parul Christian, Alemayehu Worku, Yemane Berhane, Anne C Lee

**Affiliations:** 1 Brown University Division of Biology and Medicine, Providence, Rhode Island, USA; 2 Department of Epidemiology and Biostatistics, Addis Continental Institute of Public Health, Addis Ababa, Ethiopia; 3 Department of Pediatric Newborn Medicine, Brigham and Women’s Hospital, Boston, Massachusetts, USA; 4 Amhara Public Health Institute, Bahir Dar, Ethiopia; 5 Reproductive Health, Addis Continental Institute of Public Health, Addis Ababa, Ethiopia; 6 Global Health and Health Policy, Addis Continental Institute of Public Health, Addis Ababa, Ethiopia; 7 Department of International Health, Johns Hopkins Bloomberg School of Public Health, Baltimore, Maryland, USA; 8 Harvard University, Boston, Massachusetts, USA; 9 Developmental Medicine, Boston Children’s Hospital, Boston, Massachusetts, USA; 10 Addis Continental Institute of Public Health, Addis Ababa, Ethiopia

**Keywords:** Inflammation, Maternal medicine, NUTRITION & DIETETICS, NEONATOLOGY, Genomic Medicine, Microbiota

## Abstract

**ABSTRACT:**

**Introduction:**

Maternal undernutrition and infections during pregnancy may influence birth and long-term child development outcomes. Characterising the micronutrient, metabolomic and microbiome profiles of pregnant women and infants may elucidate the underlying biology of adverse birth outcomes and early child development in the first 1000 days.

**Methods and analysis:**

The Enhancing Nutrition and Antenatal Infection Treatment (ENAT) study was a 2×2 factorial, randomised clinical effectiveness study conducted in Amhara, Ethiopia from August 2020 to June 2022. We cluster-randomised pregnant women (n=2399) to receive either a nutrition intervention (iron-folic acid (IFA), iodised salt and balanced energy-protein supplementation for women with mid-upper arm circumference <23 cm) or routine care (IFA only), and individually randomised women to an infection control intervention (genitourinary tract infection screening-treatment and screening-treatment of stool parasites) or routine care (syndromic approach). Participants were followed until 1 month postpartum. A subset of 532 women-infant dyads were consecutively enrolled in the biospecimen substudy from July 2021 to August 2022. Specimens were collected at enrolment (<24 weeks) and antenatal care follow-up (third trimester), and 1–6 months postdelivery. A subset of ENAT mother–infant dyads (n=462) was enrolled in the Longitudinal Infant Development and Growth study that followed infants until 24 months postpartum, from February 2023 to June 2024. We will determine the impact of ENAT interventions on micronutrient status, inflammation biomarkers and metabolomic and microbiome profiles. We will also determine the association of these profiles with birth outcomes and infant neurodevelopment.

**Ethics and dissemination:**

These studies were approved by the Institutional Review Boards of Addis Continental Institute of Public Health (ACIPH/IRB/002/2022) and Mass General Brigham (2023P000461). Results will be disseminated to international stakeholders via peer-reviewed journals and locally via strategic dissemination sessions.

**Trial registration numbers:**

ISRCTN15116516 and NCT06296238.

**STRENGTHS AND LIMITATIONS OF THIS STUDY**

- Data and biospecimens are from an understudied, high disease burden, rural region in Ethiopia, providing unique and novel insights into biologic mechanisms of adverse pregnancy outcomes and child development in similar settings.
- A variety of different biospecimens will provide a comprehensive profile of biological changes in women during and after pregnancy and infants in their early days.
- High-quality gestational age dating and birth weight measurement in the cohort.
- At the health centres where specimens were collected, we were not able to immediately flash freeze samples. For some samples (like stool), we used solution media to stabilise DNA/RNA and expression profiles at ambient temperature, until the samples could be frozen at the −80°C freezer at the local laboratory.
- There may be some sample-to-sample variability due to differences in day or time of sample collection, which we tried to standardise when possible (eg, first breastmilk expression of the day). Additionally, for urine iodine, we will be calculating a coefficient of variation of a 10% subset of the samples being tested.

## Introduction

 Inadequate prenatal nutrition and infection control contribute to adverse health and development outcomes in the first 1000 days of infants’ lives.^1 2^ In 2020, approximately 19.8 million babies were born with low birth weight (LBW) globally, with 27% in sub-Saharan Africa.^3^ Undernutrition in pregnancy may result in babies being born preterm (<37 weeks gestational age), with LBW (<2500 g) and/or small for gestational age (<10th percentile of the newborn size standards of the International Fetal and Newborn Growth Consortium for the 21st Century (INTERGROWTH-21st^4^)), all factors that may contribute to increased lifetime morbidity and potential neurodevelopmental delays in later years.^5^,^7^ One intervention to address these adverse outcomes is balanced energy-protein (BEP) supplementation during pregnancy for women, recommended by the World Health Organization (WHO) in regions of high population-level undernutrition. However, these recommendations were initially based on limited evidence, and the WHO recommended additional research to bridge this knowledge gap.^8^,^10^ In addition to optimised prenatal nutrition, there are potential benefits of treating genitourinary tract and helminthic infections to mitigate both adverse maternal and pregnancy outcomes related to inflammation during pregnancy.^2 8^

Ethiopia has a high population prevalence of undernutrition and infectious diseases among women of reproductive age. There are limited data on the impact of interventions to improve both prenatal nutrition and infection control on birth outcomes. To address this, our team conducted the Enhancing Nutrition and Antenatal Infection Treatment (ENAT) study in Amhara, Ethiopia. The ENAT study was a 2×2 factorial randomised clinical effectiveness study, where pregnant women were cluster-randomised to receive either nutrition intervention (including BEP) or routine care, and individually randomised to receive an infection control intervention package or routine care.^11^ The primary aims of the ENAT study were to measure the impact of maternal nutrition and infection control intervention packages on infant birth outcomes (infant weight and length). Secondary outcomes included LBW, preterm live birth, stillbirth, size-for-gestational age, newborn head circumference, maternal anaemia and maternal stress, among others. Primary results of the parent trial are forthcoming. We also conducted the Longitudinal Infant Development and Growth (LIDG) study, a longitudinal 24-month follow-up of a subset of ENAT dyads (n=462) to assess the impact of prenatal interventions on long-term child neurodevelopment among ENAT infants. The protocols of the ENAT and LIDG studies were previously published.^11 12^

In this manuscript, we detail the protocols of the ENAT–LIDG biospecimen substudy. There is limited evidence on the impact of prenatal nutrition interventions on maternal–infant biology during pregnancy, delivery and postnatally in low- and middle-income countries (LMICs). As part of a consortium of pregnancy BEP studies funded by the Gates Foundation, we will explore the impact of nutrition interventions, including BEP on multiomics profiles in pregnant women and their infants in LMICs.^13^,^15^ This substudy aimed to explore the impact of the ENAT interventions on maternal and infant metabolomic profiles, microbiome compositions, iodine and iron status, inflammation biomarkers and gut pathogens. It is crucial to understand the biological processes underlying fetal growth, preterm birth and child growth and development to develop effective interventions to mitigate maternal undernutrition and improve infant outcomes in these populations and settings.^16^,^18^ Using a variety of biospecimens (whole blood, blood serum, stool, urine and breastmilk) that were collected, we will investigate the biological mechanisms influencing molecular level changes as an effect of the prenatal ENAT interventions on birth outcomes and child growth and development until 24 months.

## Methods

### Study setting and population

The ENAT study was conducted in 12 health centres in Amhara, Ethiopia. For ENAT, pregnant women at ≤24 weeks gestation presenting for antenatal care at the study sites were enrolled in the study if they met the eligibility criteria. Women were excluded if they had a non-viable pregnancy at ≤24 weeks gestation, determined by a lack of heartbeat on the enrolment ultrasound or were unable to return for follow-up visits (typically >2 hours travel distance from health centre). Within the ENAT study, the biospecimen substudy was conducted in a subset of six health centres (three enhanced nutrition and three routine care centres) that had functional laboratory rooms and were accessible from the regional city/central laboratory (Ambesamie, Arbgebeya, Lalibela, Legdiya, Yifag and Yismala). The LIDG study enrolled the infants born to women included in these ENAT biospecimen health centres who intended to stay in the study catchment area after giving birth. Children with major congenital anomalies, severe morbidity or developmental disorders, acute symptoms of illness (eg, headache, vomiting or dizziness) or neonatal encephalopathy were excluded from the study.

### Study design

**Figure 1:**
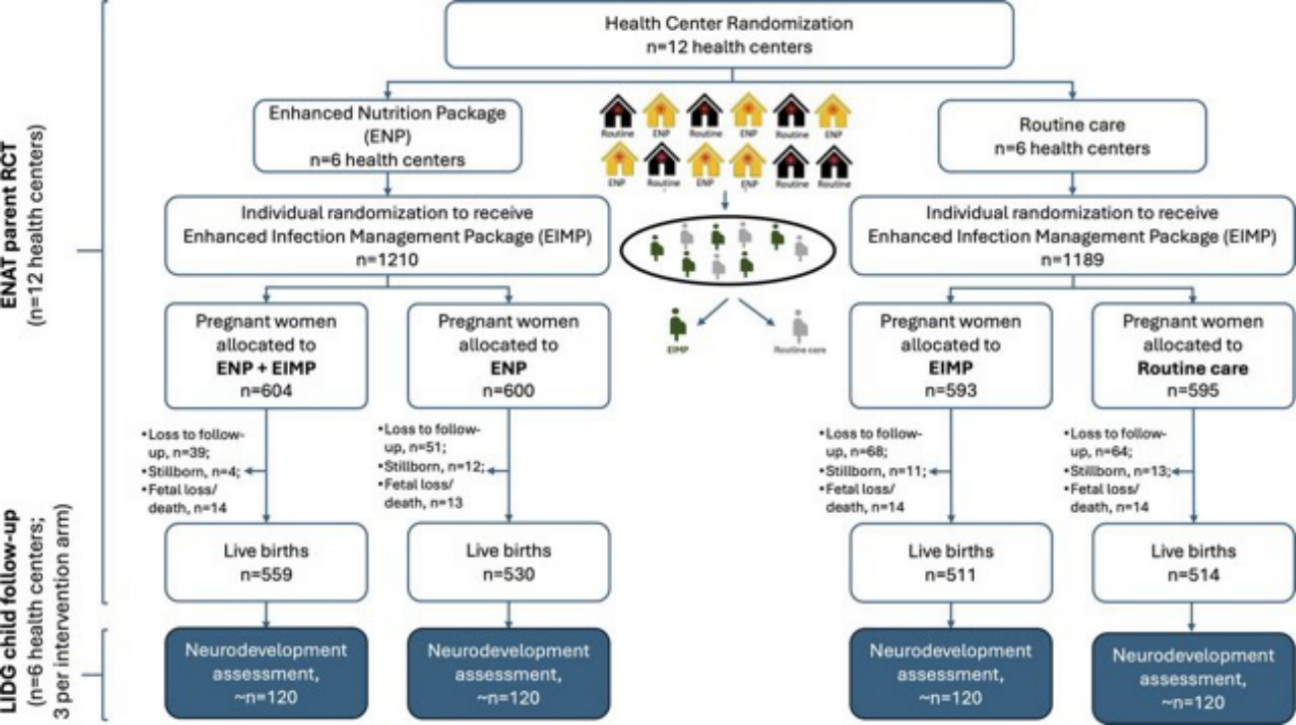
Enhancing Nutrition and Antenatal Infection Treatment (ENAT) study and Longitudinal Infant Development and Growth (LIDG) child follow-up study design participant flow. Figure from *Impact of maternal antenatal nutrition and infection treatment interventions on Longitudinal Infant Development and Growth in rural Ethiopia: protocol of the LIDG child follow-up study* by Workneh *et al* 2024. EIMP, enhanced infection management package; ENAT, Enhancing Nutrition and Antenatal Infection Treatment; ENP, enhanced nutrition package; LIDG, Longitudinal Infant Development and Growth; RCT, randomised controlled trial.

The ENAT study was a pragmatic, open-label 2×2 factorial, randomised clinical effectiveness study with cluster-randomisation of an enhanced nutrition package (ENP) intervention and individual-level randomisation of an enhanced infection management package (EIMP) intervention (figure 1). The detailed study protocol has been previously published^11^ and this protocol paper details the protocols for biospecimen collection and planned analysis.

The LIDG study was a follow-up cohort study to ENAT in which biospecimens and neurodevelopmental assessments of infants born to women in ENAT were collected/conducted to assess neurodevelopment over the first 24 months of life. Study visits for children were at approximately 12 months (enrolment), 18 months and 24 months, and included the following assessments: Hammersmith Infant Neurological Examination, eye-tracking, MRI, Bayley-III, electroencephalogram (EEG), anthropometrics, diet questionnaires, home environment, and child health assessments and biospecimens.^12^

### Study interventions

#### Enhanced Nutrition Package (ENP) Intervention

The ENP intervention consisted of (1) nutritional counselling, (2) monthly supply of high quality adequately iodised salt, (3) iron-folate (IFA) tablets and (4) women with an upper-middle arm circumference (MUAC) of <23 cm received a fortified balanced energy-protein (BEP) supplement monthly. The BEP supplement was a locally produced, micronutrient fortified, corn soya blend (World Food Programme Super Cereal). The nutrition routine care arm consisted of IFA provision.

##### Study nutrition supplements

###### Iron-folate (IFA)

Following Ethiopian Ministry of Health recommendations, the health system distributes 60 mg iron plus 400 ug folic acid supplements to be taken once daily during pregnancy to all women. Due to our formative work indicating hesitancy to take such supplements in fear of delivering ‘big babies’ and gastric irritation as a side effect,^19^ intensive counselling was provided to pregnant women in the ENP arm to encourage compliance, as well as compliance monitoring and resupply at ANC visits as needed.

###### Iodised salt

A regular monthly household supply of adequately iodised salt (Waff Manufacturing, 30–40 ppm potassium iodate, 600 g bottle) was provided to women in the ENP arm for household consumption, and they were encouraged to use approximately three pinches daily.

###### Fortified Balanced-Energy Protein (BEP)

A regular monthly supply of locally produced, micronutrient fortified, corn soya flour blend (Super Cereal, Faffa Food Share Company, Addis Ababa, Ethiopia) was provided to women in the ENP arm with a MUAC<23 cm at enrolment or any ANC visit. The composition of the BEP used is described in table 1.

**Table 1:** Super cereal corn soya blend composition (per daily 200 g serving)

Nutrient	Units	ENAT CSB content(200 gm)	Recommended target*
Energy	Kcal	760	250–500 Kcal per daily serving†
Protein	gm	28	14–18 g†
Fat	%	12	10%–60% of energy
Vitamin A	mcg RE	2076	550–770‡
Vitamin D	mcg	22.1	10–15
Vitamin E	mg	16.6	16–19
Vitamin K	mcg	60	72–90
Vitamin B_1_	mg	0.4	1.2–1.4
Vitamin B_2_	mg	2.8	1.3–1.6
Vitamin B_3_	mg	16	14–18
Folic acid	mcg	220	400–600
Vitamin B_6_	mg	2.0	1.7–2.0
Vitamin C	mg	180	100–120
Calcium	mg	724	500–1500
Iron	mg	8	22–27
Iodine	mcg	80	209–290
Phosphorous	mg	560	300–700
Zinc	mg	10	15–20

* Targets are results of an Expert Consultation held at Bill and Melinda Gates Foundation.

† Energy balance from macronutrient: portion size can be doubled in settings of high energy gaps, such as maternal malnutrition and where prevalence of birthweightbirth weight is high.

‡ Macronutrient recommendations were primarily based uponon Institute of Medicine () estimated average requirements () and recommended dietary allowances () values.

CSB, corn soya blend; ENAT, Enhancing Nutrition and Antenatal Infection Treatment.

### Enhanced Infection Management Package (EIMP) Intervention

The EIMP intervention consisted of screening and treating for urinary tract infections (UTIs), sexually transmitted infections (STIs) and reproductive tract infections (RTIs) and enhanced deworming. UTIs were treated based on sensitivity patterns. Symptomatic RTIs (eg, trichomonas, bacterial vaginosis) were diagnosed with point-of-care tests and treated accordingly with their partners. For intestinal parasites, presumptive deworming was conducted at the second trimester visit with mebendazole (500 mg). At the next antenatal care visit, approximately 1 month later, stool was screened with wet-mount microscopy and targeted treatment was provided for identified parasitic infections. The infection routine care arm involved syndromic management of maternal infections according to Ethiopian Ministry of Health recommendations.

### Biospecimen collection and processing

Maternal whole blood, serum, stool, urine, vaginal swabs and breastmilk samples were collected at trimesters 1, 2 and 3, and 1–3 months postdelivery. Umbilical cord whole blood and serum, and infant whole blood and stool were also collected at birth and 1–2 months postnatal visit(s), respectively. For samples collected during the COVID-19 pandemic, all local health and prevention protocols and guidance were followed to collect and process biospecimens. Specimens were collected at each study health centre by trained laboratory technicians assisted by study nurses and transported using triple packaging containing ice packs within 48 hours of collection to the Amhara Public Health Institute (APHI) central laboratory for processing and longer storage at −80°C. A comprehensive list and timing of biospecimens collected in this study are shown in table 2.

**Table 2:** ENAT study visits and biospecimen collection

Biospecimen type (collection mechanisms)	Enrolment visit (<24 week)	ANC follow-up visit (~30–34 weeks)	Birth (within 72 hours of birth)	Postnatal follow-up visit (1–6 months)*
Urine	M	M		
Vaginal swab	M	M		
Serum	M	M	C	
Whole blood (DBS)	M	M	C	I
Whole blood (VAMS)	M	M	C	I
Stool (Genotek)	M	M		M, I
Stool (TaqMan)	M	M		M, I
Breastmilk				M

* Postnatal visits were stopped prematurely due to conflict in the study region.

ANC, antenatal care; C, cord; DBS, dried blood spot; ENAT, Enhancing Nutrition and Antenatal Infection Treatment; I, infant; M, maternal; VAMS, volumetric absorptive microsampling.

#### Maternal urine

Participants were provided with detailed instructions on how to collect the proper sample. Mid-stream clean catch urine samples were obtained from the participants at the study health centre using sterile urine collection cups. The collected samples were then transferred into 10 mL boric acid vacutainers for transport until urine culture was processed at APHI for the main study. The samples were aliquoted into 5 mL sterile cryotubes for storage at APHI for future analysis. Additionally, UTI testing was done with urine culture, and antibiotic susceptibility testing was conducted using Vitek cards.

#### Maternal vaginal swab

Participants were instructed by the health centre laboratory technicians and study nurses on how to self-collect mid-vaginal swab samples. Formative work was conducted prior to the study regarding the self-collection of swabs, and it was both highly acceptable (96%) and high-quality. Based on this formative work, sample collection instructions were revised to improve quality. Following handwashing with soap, women used nylon swabs to swab their vagina and placed them on a specimen collection tray. Two swabs were collected. STIs were detected using self-collected vaginal swabs tested for gonorrhoea and chlamydia with the GeneXpert platform. The laboratory technicians then transferred the remaining swabs into the DNA/RNA shield collection tubes, securely capped them and gently inverted the tubes several times (ensuring no foaming). Samples were transported to the APHI and aliquoted into sterile cryotubes and stored at −80°C.

#### Maternal blood

##### Serum

Trained laboratory technicians performed venipuncture to collect a total of 7 mL of whole blood from participants during each visit. The blood was drawn into a yellow-top serum separator tube (SST) and kept upright at room temperature until transport to APHI. On arrival at APHI, the SST tube was centrifuged at varying speeds (5, 10 or 15 000 RPM) to separate the serum from the sediment layers. The top serum layer was then carefully aliquoted in up to two sterile cryovials using a micro-pipette and stored at −80°C.

##### Dried blood spots (DBS)

Maternal prenatal DBS samples were collected from women while they were providing blood for routine laboratory testing (haemoglobin/HIV/syphilis) in trimester 1. Samples from trimesters 2 and 3 were collected via finger-stick. After the blood was collected into EDTA tubes, the tubes were gently inverted 8–10 times. Using a micropipette, 50–80 µL of blood was placed on a single spot on a Whatman 903 Proteinsaver Card (Cytiva, US), and this process was repeated three more times for a total of four spots. The Whatman cards were air-dried for 24 hours at the study health centre laboratory and placed in an individual zippered plastic bag with a humidity indicator and 2–3 small silica gels. At APHI, approximately 20 DBS samples were stored within a larger zippered bag with silica gel pillows, humidity indicators and stored at −80°C.

##### Volumetric Absorptive Microsamples (VAMS)

Whole blood samples were collected using a volumetric absorptive microsampling (VAMS) device, MitraTM by Neoteryx (Torrance, CA, USA) from women while they were providing blood for routine laboratory testing by collecting additional drops, directly after collecting DBS samples (described previously). A total of 40 µL (4×10 µL) of blood was collected from the women at each timepoint by lightly touching the blood sample with the Mitra tip until it absorbed the required volume. In the absence of routine ANC phlebotomy in trimesters 2 and 3, whole blood was collected by the finger stick technique, with the Mitra tips hovering above the finger (without direct contact) until the tip turned fully red. Samples were air-dried either in their clamshell or over an empty 96-well collection plate for a minimum of 1 hour (max 3 hours). After drying, stored in Mitra autoracks (96-Sampler, item number: 108, Neoteryx, Torrance, CA, USA), wrapped in aluminium foil, placed in zippered bags with desiccant and transported to the APHI where they were transferred to a separate cryovial and stored at −80°C.

### Maternal stool

Participants were instructed by the health centre laboratory techs and study nurses on how to collect stool samples. Women were instructed to urinate into the toilet before collecting stool in the study-provided container while avoiding urine, soil, detergent, fragrance or water contaminating the sample. The laboratory tech evaluated the stool sample, completed the Bristol Stool Chart and measured the pH using nitrazine pH paper. Samples were then homogenised by using a plastic spoon and stirring the sample. Stool samples were aliquoted for Genotek analysis using an OMNIgene. GUT collection kit for TaqMan analysis was collected using a DNA/RNA shield tube. All stool samples were refrigerated at the local health centre after collection and transported to APHI. At APHI, Genotek samples were aliquoted into two cryovials and were stored at −80°C. TaqMan samples were first stored in the DNA/RNA shield tube, and additional stool was aliquoted into three cryovials and stored at −80°C.

### Infant blood

#### Umbilical cord and infant dried blood spots (DBS)

DBS samples were collected from the umbilical cord after delivery and from infants at 1–3 months. After delivery of the infant and placenta, the umbilical cord was double-clamped near the placenta and baby, cut and wiped with clean and dry gauze to remove maternal blood and contaminants. Umbilical cord blood was then drawn using an 18-gauge needle mounted on a 10 mL syringe with a luer tip. Four separate blood spots were then carefully dispensed onto a Whatman card. For infants, whole blood was collected by the heel stick technique. Samples were air-dried, transported and stored using the same procedures as maternal DBS samples.

### Infant stool

Mothers were instructed to wipe down the infant’s groin and public area with wipes provided by the study. Without applying any lotions or ointments, mothers lay the infant on the diaper or a cloth and stimulated the anal region using a wet cotton bud. Once the infant had a bowel movement, mothers collected the stool sample following the same instructions as their own samples (described earlier). Samples were processed, transported and stored using the same procedures as maternal stool samples.

A copy of the samples was shipped from Ethiopia to Boston, MA in the USA on dry ice and with temperature monitors for temporary storage and testing/analysis as previously described. Any samples with a single copy were retained in Ethiopia in compliance with Ethiopian regulatory requirements.

### Planned laboratory analyses

We intend to approach the analyses of our collected biospecimens using a coordinated multiomics approach to characterise the metabolome and microbiome of maternal and infant whole blood, serum and stool during pregnancy and in the postnatal period.^17^ Certain analyses will be coordinated with other prenatal BEP supplementation trials. Additionally, we will conduct analyses of certain biospecimens for micronutrient concentrations and inflammation biomarkers. Data from maternal urine and vaginal swabs will also provide insights into biological changes within women during pregnancy and breastfeeding. A comprehensive table of planned analyses is presented in table 3.

**Table 3:** Summary of analyses to be performed on biospecimens

Biospecimen	Collection device	Planned testing	Analytic technique	Testing laboratory
*Maternal samples*				
Maternal whole blood	Whatman Card (DBS)	Thyroid function (TSH, T4, T3, TG) andleucocyte telomere length	ELISAs (eg, DBS-Tg assay) qPCR	Human Nutrition Lab, Zurich, SwitzerlandDrury Lab at Boston Children’s Hospital, Boston, MA, USA
Maternal whole blood	VAMS	Metabolomics	rLC-MS	Sapient Bioanalytics, San Diego, CA, USA
Maternal serum	EDTA tube	Ferritin, Soluble transferrin receptor (sTfR), total body iron, Hepcidin	ELISAs	TBD
Maternal stool	Genotek: OMNIgene.GUT	Microbiome profiling	Shotgun metagenomics	University of Wisconsin-Madison, WI, USA
Maternal stool	TaqMan: DNA/RNA shield tube	Enteropathogen profiling	qPCR	University of Virginia, VA, USA
Maternal urine	Poly tube	TSH, Tg, fT4, CRP, AGP, IL-6, IL-6R and iodine concentrations	ELISAs	Ethiopia Public Health Institute, Addis Ababa, Ethiopia
Maternal vaginal swab	Swab	Microbiome profiling	Shotgun metagenomics	TBD
*Cord/infant samples*				
Umbilical cord blood spot	Whatman card (DBS)	Metabolomics CRP, AGP, IL-6, IL-6R, TSH, Tg, fT4,	rLC-MS	Sapient Bioanalytics, San Diego, CA, USATBD
Infant blood	Whatman card (DBS)	Metabolomics andleucocyte telomere length	rLC-MS qPCR	Sapient Bioanalytics, San Diego, CA, USA Drury Laboratory at Boston Children’s Hospital, Boston, MA, USA
Infant stool	Genotek: OMNIgene.GUT	Microbiome profiling	Shotgun metagenomics	University of Wisconsin-Madison, WI, USA
Infant stool	TaqMan: DNA/RNA shield tube	Enteropathogen profiling	qPCR	University of Virginia, VA, USA

AGP, α1-acid glycoprotein; CRP, C-reactive protein; DBS, dried blood spot; EDTA, ethylenediaminetetraacetic acid; ELISA, enzyme-linked immunosorbent assay; qPCR, Quantitative Polymerase Chain Reaction; rLC-MS, rapid liquid chromatography–mass spectrometry; TBD, to be decided; VAMS, volumetric absorptive microsampling.

#### Inflammation

Maternal diet and infections during pregnancy both may influence inflammatory processes and impact the development and growth of the fetus and infant.^20 21^ Using infant and maternal DBS, we will examine the effects of the ENAT interventions on maternal and newborn inflammation biomarkers including C-reactive protein (CRP), α1-acid glycoprotein (AGP), interleukin-6 and 6R (IL-6 and IL-6R) and investigate the association of maternal and newborn inflammation on long-term neurodevelopment in infants.

#### Micronutrients

##### Iodine status

Iodine is required for the synthesis of thyroid hormones, which are essential for human brain development, and iodine supplementation during pregnancy has been shown to mitigate the adverse effects of iodine deficiency.^22^ Using banked samples, we will test urinary iodine concentration at the Ethiopia Public Health Institute (Addis Ababa, Ethiopia) to determine the impact of ENAT’s intensive iodised salt intervention component during pregnancy on infant neurodevelopment and thyroid function. DBSs will also be tested for thyroid function markers (TSH, Free T4, Thyroglobulin) at the Human Nutrition Laboratory (Zurich, Switzerland). Of the samples being tested for urine iodine, a subset of 10% will be retested and we will calculate a coefficient of variation to determine any sample-to-sample variability.

##### Iron status

We will analyse maternal serum for hepcidin, ferritin and soluble transferrin receptors to assess iron status. Total body iron will be calculated, and adjustments will be made for inflammation biomarkers (CRP and AGP) as recommended by the BRINDA project.^23^ The impact of the ENAT intervention on iron status will be evaluated, and associations between maternal prenatal iron status and child development outcomes will be examined. Finally, we will preserve blood samples for further testing of other micronutrients, such as selenium.^24^

### Metabolomics

Metabolomics is the systematic analysis of human biofluids, such as blood, to describe the composition and metabolic changes within the human body at a specific time point. It is essential to understand the biological processes and pathways influenced by interventions involving biological elements like supplements and antibiotics. Using maternal and infant whole blood samples collected across multiple time points via VAMS and DBS, we will conduct untargeted metabolomics analysis using rapid liquid chromatography-mass spectroscopy (rLC-MS) at Sapient Analytics (California, USA).^25^ Internal control samples will be used to standardise testing and counteract potential batch effects. This discovery work will help us to identify metabolites both in pregnant women and infants that are associated with the ENAT interventions, as well as adverse birth outcomes such as preterm birth, small-for-gestational-age birth and LBW.

### Microbiome profiling

Multiple studies have established the importance of the maternal microbiome on infant birth and growth outcomes; however, there is limited data on the effects of intervention during pregnancy.^26^ Using stool samples collected via OMNIgene.GUT collection kits, we will profile the maternal and infant gut microbiomes through shotgun metagenomic sequencing at the University of Wisconsin (Madison, WI, USA). This approach will accurately measure the richness and abundance of microbes and the differences between those receiving the ENAT nutrition intervention, infection intervention, both or neither. Additionally, we will also investigate how the maternal microbiome during pregnancy may impact infant birth and neurodevelopmental outcomes. This analysis will be conducted in collaboration with the Sonnenberg Laboratory at Stanford University (Stanford, CA, USA).

### Gut enteropathogens

It has been hypothesised that enteropathogen infection may cause intestinal inflammation and contribute to stunting and micronutrient deficiencies in infants.^13^ Stool samples collected in DNA/RNA shield will be sent to the University of Virginia for testing using custom-designed TaqMan array cards (TAC). We will determine the pathogenic burden within pregnant women. We will also examine the impact of the ENAT deworming interventions on stool pathogens.

### Telomeres

Leucocyte telomere length will be determined from maternal and cord blood DBS DNA. DNA will be extracted from the samples using the Qiagen Qiamp Blood Mini Kit protocol at the Drury Laboratory at Boston Children’s Hospital (Boston, MA, USA). Telomere length will be assayed following a standardised protocol.^27^

### Statistical analyses

We will have two different approaches to our analyses. In the primary analysis, we will determine differences in biomarkers/profiles between the randomised study intervention arms, and in the secondary analysis, we will determine the associations of biomarkers with adverse outcomes (pregnancy or child developmental outcomes).

#### Intervention effects

In our primary analysis, we will examine differences in biomarkers or ’omics profiles across the randomised intervention study arms. We will use intention-to-treat as the primary analyses where individuals are analysed according to their originally assigned study arm, and in secondary analysis, we will also explore effects of individual interventions (ie, BEP) and adherence/dose. Outcomes will be compared between the primary factors (ENP or EIMP) to determine their marginal effects (ENP vs not-ENP, and EIMP vs not-EIMP). We will estimate differences in biomarkers, using linear mixed regression models. Potential confounding bias should be minimised due to the randomised study design; however, we will assess group differences in demographic and clinical characteristics at both cluster- and individual randomisation levels, as recommended by Consolidated Standards of Reporting Trials.^28^ Baseline covariate imbalances associated with both the outcome and exposure status (p<0.10) will be included in adjusted models as potential confounders. Multiple births will be excluded from analyses using birth outcomes due to the known increased rate of preterm birth and growth restriction among multiple deliveries. When large volumes of tests are conducted for metabolomics or genomics analyses, we will use the Benjamini-Hochberg method to correct for multiple testing, as appropriate.

#### Adverse pregnancy/child development outcomes

In our secondary analyses, we will examine the associations of biomarkers or ’omics profiles and adverse birth outcomes (eg, preterm birth, stillbirth and growth restriction) or child development outcomes (eg, EEG outcomes, MRI outcomes, eye tracking, etc). In most of these analyses, we will use a prospective cohort study design and estimate differences among birth or child development outcomes using linear or logistic regression models (based on the outcome), and adjust for covariates as relevant for specific outcomes. Details for each individual relationship examined will be included in the publications for each individual analysis.

### Patient and public involvement

The community was involved in the design of the parent ENAT study. We conducted formative research including in-depth interviews with patients and community members regarding the feasibility and acceptability of interventions as well as specific sample type collection procedures, including urine and vaginal swabs. Results of this study will be disseminated to the Ministry of Health and peer-reviewed journal publications.

## Metadata

We collected demographic, lifestyle, nutrition, and health data from all study participants along with birth outcomes of the infants using Survey Solutions by the World Bank. Full details on data collection procedures have been previously described.^11^

## Data quality

Study staff collected data directly on study-provided tablets using Survey Solutions (V.20.8.2 (build 27 880)) and synchronised when possible, to ACIPH’s local server. Internet disruptions occasionally interfered with this schedule. Weekly data checks were conducted by the data team, and missingness or inconsistent data were highlighted to the field team to be addressed. All laboratory testing and data being generated are/will be conducted in collaboration with reputable laboratories that have previously conducted similar analyses.

## Ethics and dissemination

All participants consented to this study prior to participation, initiation of study procedures or biospecimen collection. Only deidentified data will be shared with laboratories conducting testing. This study was approved by the Addis Continental Institute of Public Health (001-A12019) and the Mass General Brigham Institutional Review Board (2018P002479). The Amhara Public Health Institute granted local permission to conduct the study and permissions were obtained from district health offices and the participating study health centres. All peer-reviewed publications will only contain deidentified data.
