# Supplementary material for: Investigating biological mechanisms of adverse birth outcomes and early child development in Amhara, Ethiopia: protocol of biospecimen collection and analysis of the Enhancing Nutrition and Antenatal Infection Treatment (ENAT) randomised effectiveness study
Source: BMJ Open. 2025 Apr 28;15(4):e098686. doi: 10.1136/bmjopen-2024-098686 (PMC12039041; doi:10.1136/bmjopen-2024-098686)
Supplement: Supplementary file 1 [file bmjopen-2024-098686.reviewer_comments.pdf]

PEER REVIEW HISTORY

BMJ Open publishes all reviews undertaken for accepted manuscripts. Reviewers are asked to complete a checklist review form and are provided with free text boxes to elaborate on their assessment. These free text comments are reproduced below.

ARTICLE DETAILS

Title (Provisional)

Investigating Biological Mechanisms of Adverse Birth Outcomes and Early Child Development in Amhara, Ethiopia: Protocol of Biospecimen Collection and Analysis of the Enhancing Nutrition and Antenatal Infection Treatment (ENAT) Randomized Effectiveness Study

Authors

Roy Paladhi, Unmesha; Workneh, Firehiwot; Baye, Estifanos; Derebe, Mulatu Melese; Yibeltal, Kalkidan; Fasil, Nebiyou; Driker, Sophie; Van Dyk, Fred; Chin, Theresa Inez Inez; North, Krysten; Jensen, Sarah K. G.; Christian, Parul; Worku, Alemayehu; Berhane, Yemane; Lee, Anne C

VERSION 1 - REVIEW

**Reviewer**	**1**
**Name**	**Lee, Gwenyth**
**Affiliation**	**Rutgers School of Public Health, Epidemiology**
**Date**	**27-Feb-2025**
**COI**	**None**

This protocol paper describes biospecimen collection and planned biospecimen analysis for samples from the ENAT study, an RCT designed to evaluate the impact of nutritional and infection control interventions in the 2nd-3rd trimester of pregnancy on infant birth outcomes and development. A large variety of biospecimens were collected, and many laboratory analyses are planned. These are clearly laid out, although with a limited amount of detail. However, there is relatively little emphasis on plans for statistical analysis, which creates the overall impression that the project is more exploratory, probably, than it truly is. I would therefore recommend expanding this section.

The two limitations listed in the 'strengths and limitations' section, although valid, do not seem to be the most significant, nor are they the limitations that are most related to the sample collection and sample analysis, which is the focus of the paper. For example, surprisingly little is said about how methods of field collection and storage that might affect

particular assay results, or how sample-to-sample variability in some assays limit the extent to which results from a single sample can be generalized.

Line 186 – Could the authors clarify how the 10g of salt daily that the study team encouraged participating women to consume relates to the recommended sodium intake- was there any risk of women in the intervention arm consuming too much salt?

Line 203-205. Could the authors mention or perhaps just further describe and interpret the Ethiopian MOH recommendations for deworming in pregnancy? Presumptive deworming is not effective for all helminth infections, and the safety and efficacy of other treatments in pregnancy has been questioned, so it would be helpful to understand a little more about the protocol for targeted treatment for infections identified after the presumptive deworming.

Infant stool is described in the planned analysis but collection seems to be absence from section 4.

How much time passed between defecation and stool preservation in the OMNIgene gut collection kit and the DNA/RNA shield?

Was hemoglobin also measured at the time of blood collection?

**Reviewer**	**2**
**Name**	**Lelijveld, Natasha**
**Affiliation**	**London School of Hygiene & Tropical Medicine**
**Date**	**12-Mar-2025**
**COI**	**None**

A very well-written and thorough protocol for an interesting study. I just have a few small comments

1. In the 'study strengths and limitations' section at the start of the study you mention transportation difficulties as a limitation, however this point is not made in the main manuscript. It feels like this section should represent information that is related to the rest of the article, so could something be added to the methods section? or perhaps this limitations section could be amended to something else relevant?

2. There is no mention of your planned statistical analyses and comparisons. It is important to document these plans to avoid 'data mining' or changing analysis plans if findings are not as expected.

2a. related to this, I wondered if you would have enough of a sample size to do a sub-analysis on adolescent mothers? Just a suggestion for your consideration.

VERSION 1 - AUTHOR RESPONSE

Reviewer #1: Dr. Gwenyth Lee, Rutgers School of Public Health

1) **This protocol paper describes biospecimen collection and planned biospecimen analysis for samples from the ENAT study, an RCT designed to evaluate the impact of nutritional and infection control interventions in the 2nd-3rd trimester of pregnancy on infant birth outcomes and development. A large variety of biospecimens were collected, and many laboratory analyses are planned. These are clearly laid out, although with a limited amount of detail. However, there is relatively little emphasis on plans for statistical analysis, which creates the overall impression that the project is more exploratory, probably, than it truly is. I would therefore recommend expanding this section.**

Thank you for your comments and the suggestion to add additional statistical analysis details. While we have limited word count and are unable to provide the detailed statistical analyses for each sample type, we have added a "Statistical Analyses" section and detailed the ENAT intervention impacts analyses (lines 429-459). It reads:

"We will have two different approaches to our analyses. In the primary analysis, we will determine differences in biomarkers/profiles between the randomized study intervention arms, and in the secondary analysis, we will determine the associations of biomarkers with adverse outcomes (pregnancy or child developmental outcomes).

6.1 Intervention Effects
In our primary analysis we will examine differences in biomarkers or 'omics profiles across the randomized intervention study arms. We will use intention-to-treat as the primary analyses where individuals are analyzed according to their originally assigned study arm, and in secondary analysis we will also explore effects of individual interventions (ie. BEP) and adherence/dose. Outcomes will be compared between the primary factors (ENP or EIMP) to determine their marginal effects (ENP versus not-ENP, and EIMP versus not-EIMP). We will estimate differences in biomarkers, using linear mixed regression models. Potential confounding bias should be minimized due to the randomized study design; however, we will assess group differences in demographic and clinical characteristics at both cluster- and individual randomization-levels, as recommended by CONSORT.[28] Baseline covariate imbalances associated with both the outcome and exposure status ($p < 0.10$) will be included in adjusted models as potential confounders. Multiple births will be excluded from analyses using birth outcomes due to the known increased rate of preterm birth and growth restriction among multiple deliveries. When large volumes of tests are conducted for metabolomics or genomics analyses, we will use the Benjamini-Hochberg method to correct for multiple testing, as appropriate.

6.2 Adverse Pregnancy/Child Development Outcomes
In our secondary analyses we will examine the associations of biomarkers or 'omics profiles and adverse birth outcomes (eg. preterm birth, stillbirth, growth restriction) or child development outcomes (eg. EEG outcomes, MRI outcomes, eye tracking, etc.). In most of these analyses, we will use a prospective cohort study design and estimate differences among birth or child development outcomes using linear or logistic regression models (based on the outcome), and adjust for covariates as relevant for specific outcomes. Details for each individual relationship examined will be included in the publications for each individual analyses."

2) **The two limitations listed in the 'strengths and limitations' section, although valid, do not seem to be the most significant, nor are they the limitations that are most related to the sample**

collection and sample analysis, which is the focus of the paper. For example, surprisingly little is said about how methods of field collection and storage that might affect particular assay results, or how sample-to-sample variability in some assays limit the extent to which results from a single sample can be generalized.

Thank you for these comments. We have removed the current limitations and added the following:

- *At the health centers where specimens were collected, we were not able to immediately flash freeze samples. For some samples (like stool), we used solution media to stabilize DNA/RNA and expression profiles at ambient temperature, until the samples could be frozen at -80C freezer at the local laboratory.*
- *There may be some sample-to-sample variability due to differences in day or time of sample collection which we tried to standardize as possible (eg. first breastmilk expression of the day). Additionally, for urine iodine, we will be calculating a coefficient of variation of a 10% subset of the samples being tested.*

3) **Line 186 – Could the authors clarify how the 10g of salt daily that the study team encouraged participating women to consume relates to the recommended sodium intake- was there any risk of women in the intervention arm consuming too much salt?**

We were not attempting to limit or increase sodium intake as part of the parent ENAT interventions but rather providing high quality, **adequately iodized** salt that was intended to replace their current household supply. If women finished their allocation, we provided them with an additional supply. We had operationalized the suggested household salt intake as 3 pinches of salt daily which is approximately 6g. 6g of salt contains approximately 2325mg of sodium, which is well below the daily recommended sodium intake of 6000mg for an average household of 4 (individually 1500mg). We have updated the manuscript to remove the 10g unit of measurement and leave the 3 pinches as the unit of measurement.

4) **Line 203-205. Could the authors mention or perhaps just further describe and interpret the Ethiopian MOH recommendations for deworming in pregnancy? Presumptive deworming is not effective for all helminth infections, and the safety and efficacy of other treatments in pregnancy has been questioned, so it would be helpful to understand a little more about the protocol for targeted treatment for infections identified after the presumptive deworming.**

We understand the need to clarify the exact the Ethiopian MOH recommendations. We have clarified the deworming schedule during the biospecimen collection dates and added the following language to the manuscript (lines 26-230):

"For intestinal parasites, presumptive deworming was conducted at the second trimester visit with mebendazole (500mg). At the next antenatal care visit, approximately one month later, stool was screened with wet-mount microscopy and targeted treatment was provided for identified parasitic infections."

5) **Infant stool is described in the planned analysis but collection seems to be absence from section 4.**

Thank you for highlighting this omission, We have added an "Infant Stool" subsection under the Collection section (lines 333-339). It reads:

"Mothers were instructed to wipe down the infant's groin and public area with wipes provided by the study. Without applying any lotions or ointments, mothers lay the infant on the diaper or a cloth and stimulated the anal region using a wet cotton bud. Once the infant had a bowel movement, mothers collected the stool sample following the same instructions as their own samples (described earlier). Samples were processed, transported, and stored using the same procedures as maternal stool samples."

6) **How much time passed between defecation and stool preservation in the OMNIgene gut collection kit and the DNA/RNA shield?**

   Stool samples were refrigerated within 2 hours of collection and preserved within 48 hours of collection. We have added this to the "Maternal Stool" methods section (lines 317-321):

   "All stool samples were refrigerated at the local health center after collection and transported to APHI. At APHI, Genotek samples were aliquoted into two cryovials and were stored at −80°C. TaqMan samples were first stored in the DNA/RNA shield tube and additional stool was aliquoted into three cryovials and stored at −80°C".

   Additionally, all samples were *"transported using triple packaging containing ice packs within 48 hours of collection to the Amhara Public Health Institute (APHI) central lab for processing and longer storage at −80°C"* and this has been added to the protocol (lines 241-243).

7) **Was hemoglobin also measured at the time of blood collection?**

   Maternal hemoglobin was supposed to be measured at each timepoint blood was obtained during the course of the study. However, actual collection was low due to lack of supplies, and therefore we chose to exclude this from the protocol paper as there is insufficient data for analysis.

Reviewer #2: Dr. Natasha Lelijveld, London School of Hygiene & Tropical Medicine

A very well-written and thorough protocol for an interesting study. I just have a few small comments

Thank you for your review and comments.

1) **In the 'study strengths and limitations' section at the start of the study you mention transportation difficulties as a limitation, however this point is not made in the main manuscript. It feels like this section should represent information that is related to the rest of the article, so could something be added to the methods section? or perhaps this limitations section could be amended to something else relevant?**

   Due to suggestions made by Reviewer #1 about this not being a true limitation, we have removed this from the limitation section and replaced this with language on potential limited sample-to-sample variability (and added this language to the main manuscript text):

   - *At the health centers where specimens were collected, we were not able to immediately flash freeze samples. For some samples (like stool), we used solution media to stabilize DNA/RNA and expression profiles at ambient temperature, until the samples could be frozen at -80C freezer at the local laboratory.*
   - *There may be some sample-to-sample variability due to differences in day or time of sample collection which we tried to standardize as possible (eg. first breastmilk expression of the*

day). Additionally, for urine iodine, we will be calculating a coefficient of variation of a 10% subset of the samples being tested.

2) **There is no mention of your planned statistical analyses and comparisons. It is important to document these plans to avoid 'data mining' or changing analysis plans if findings are not as expected.**
   a. **related to this, I wondered if you would have enough of a sample size to do a sub-analysis on adolescent mothers? Just a suggestion for your consideration.**

Thank you for highlighting these as an important aspect of the paper. Your comment was similar to a comment by Reviewer #1 so we have added a "Statistical Analyses" section and detailed the ENAT intervention impacts analyses (lines 429-459). It reads:

"We will have two different approaches to our analyses. In the primary analysis, we will determine differences in biomarkers/profiles between the randomized study intervention arms, and in the secondary analysis, we will determine the associations of biomarkers with adverse outcomes (pregnancy or child developmental outcomes).

6.1 Intervention Effects
In our primary analysis we will examine differences in biomarkers or 'omics profiles across the randomized intervention study arms. We will use intention-to-treat as the primary analyses where individuals are analyzed according to their originally assigned study arm, and in secondary analysis we will also explore effects of individual interventions (ie. BEP) and adherence/dose. Outcomes will be compared between the primary factors (ENP or EIMP) to determine their marginal effects (ENP versus not-ENP, and EIMP versus not-EIMP). We will estimate differences in biomarkers, using linear mixed regression models. Potential confounding bias should be minimized due to the randomized study design; however, we will assess group differences in demographic and clinical characteristics at both cluster- and individual randomization-levels, as recommended by CONSORT.[28] Baseline covariate imbalances associated with both the outcome and exposure status ($p < 0.10$) will be included in adjusted models as potential confounders. Multiple births will be excluded from analyses using birth outcomes due to the known increased rate of preterm birth and growth restriction among multiple deliveries. When large volumes of tests are conducted for metabolomics or genomics analyses, we will use the Benjamini-Hochberg method to correct for multiple testing, as appropriate.

6.2 Adverse Pregnancy/Child Development Outcomes
In our secondary analyses we will examine the associations of biomarkers or 'omics profiles and adverse birth outcomes (eg. preterm birth, stillbirth, growth restriction) or child development outcomes (eg. EEG outcomes, MRI outcomes, eye tracking, etc.). In most of these analyses, we will use a prospective cohort study design and estimate differences among birth or child development outcomes using linear or logistic regression models (based on the outcome), and adjust for covariates as relevant for specific outcomes. Details for each individual relationship examined will be included in the publications for each individual analyses."

Lastly, unfortunately, we do not have sufficient sample size to conduct a sub-analysis on adolescent mothers.
